# A phase I study of the WT2725 dosing emulsion in patients with advanced malignancies

**DOI:** 10.1038/s41598-021-01707-3

**Published:** 2021-11-16

**Authors:** Siqing Fu, David E. Piccioni, Hongtao Liu, Rimas V. Lukas, Santosh Kesari, Dawit Aregawi, David S. Hong, Kenichiro Yamaguchi, Kate Whicher, Yi Zhang, Yu-Luan Chen, Nagaraju Poola, John Eddy, David Blum

**Affiliations:** 1 Department of Investigational Cancer Therapeutics, The University of Texas MD Anderson Cancer Center, 1515 Holcombe Boulevard, Houston, TX 77030 USA; 2 UCSD Moores Cancer Center, San Diego, CA USA; 3 University of Chicago Medical Center, Chicago, IL USA; 4 Northwestern University, Chicago, IL USA; 5 Lou and Jean Malnati Brain Tumor Institute, Chicago, IL USA; 6 Saint John’s Cancer Institute and Pacific Neuroscience Institute, Santa Monica, CA USA; 7 Penn State Milton S. Hershey Medical Center, Penn State College of Medicine, Hershey, PA USA; 8 Sumitomo Dainippon Pharma Co., Ltd., Tokyo, Japan; 9 Sunovion Pharmaceuticals Inc., Marlborough, MA USA; 10 Present Address: Otsuka Pharmaceuticals, Princeton, NJ USA; 11 Present Address: Morphic Therapeutic, Waltham, MA USA

**Keywords:** Cancer immunotherapy, CNS cancer

## Abstract

WT2725 is a Wilms’ tumor gene 1 (WT1)-derived-oligopeptide vaccine designed to induce WT1-specific cytotoxic T-lymphocytes against WT1^+^ tumors in human leukocyte antigen (HLA)-A*0201^+^ and/or HLA-A*0206^+^ patients. Here, we report the results of a phase I study of WT2725. In this phase I, open-label, dose-escalation and expansion two-part study, the WT2725 dosing emulsion was administered as a monotherapy to patients with advanced malignancies known to overexpress WT1, including glioblastoma. In part 1, 44 patients were sequentially allocated to four doses: 0.3 mg (n = 5), 0.9 mg (n = 5), 3 mg (n = 6), and 9 mg (n = 28). In part 2, 18 patients were allocated to two doses: 18 mg (n = 9) and 27 mg (n = 9). No dose-limiting toxicities were observed, so the maximum tolerated dose was not reached. Median progression-free survival was 58 (95% confidence interval [CI] 56–81) days (~ 2 months) across all patients with solid tumors; median overall survival was 394 days (13.0 months) (95% CI 309–648). Overall immune-related response rate in solid tumor patients was 7.5% (95% CI 2.6–19.9); response was most prominent in the glioblastoma subgroup. Overall, 62.3% of patients were considered cytotoxic T-lymphocyte responders; the proportion increased with increasing WT2725 dosing emulsion dose. WT2725 dosing emulsion was well tolerated. Preliminary tumor response and biological marker data suggest that WT2725 dosing emulsion may exert antitumor activity in malignancies known to overexpress the WT1 protein, particularly glioblastoma, and provide a rationale for future clinical development.

**Trial registration:** NCT01621542.

## Introduction

WT2725 is a Wilms’ tumor gene 1 (WT1)-derived-oligopeptide vaccine designed to induce WT1-specific cytotoxic T-lymphocytes (CTLs) against WT1^+^ tumors in human leukocyte antigen (HLA)-A*0201^+^ and/or HLA-A*0206^+^ patients.

*WT1* encodes a zinc-finger transcription factor that is involved in cell proliferation, differentiation, apoptosis, and organ development, and is overexpressed in various malignancies^1–4^. *WT1*/WT1 is expressed/overexpressed in most (63–94%) glioblastoma samples, most (80–90%) acute myeloid leukemia (AML) patients, 96% of non-small-cell lung carcinoma (NSCLC) samples in one study (with expression markedly higher than in normal tissues across multiple studies), and 56–71% of ovarian cancers (when evaluated as a single set; 50–100% of serous carcinomas and 0–13% of non-serious ovarian tumors)^5–22^. Data demonstrate that WT1-derived peptides can trigger cellular and humoral immune responses in vivo; WT1-specific CTLs can lyse WT1-expressing tumor cells without harming normal tissue^1^. Following vaccination, WT2725 (a synthetic WT1 peptide) has potential to stimulate the host immune system to induce a CTL response against cancer cells that overexpress the WT1 protein, leading to cell lysis and preventing further tumor cell proliferation.

Previously studied WT1 vaccines have induced immunogenicity and antitumor responses in clinical trials of various malignancies^2,23–36^. However, injection of peptides in aqueous solutions alone is not always effective in stimulating a CTL response; different adjuvants can affect the body’s immune response to peptide antigens. For example, water-in-oil (W/O) emulsions enhance the immunogenicity of antigens by creating a depot effect that prevents the antigen from accessing tissue and blood-borne proteases but facilitates translocation into antigen-presenting cells^37^.

The WT2725 dosing emulsion comprises WT2725 (the acetate salt of a synthetic peptide with the same sequence as the naturally occurring peptide WT1_126–134_) diluted in a peptide-diluting solution, for administration with a novel W/O pre-emulsion adjuvant. In vitro studies using human peripheral blood mononuclear cells from an HLA-A*0201^+^ healthy donor confirmed that the WT2725 peptide binds to HLA-A*0201 (antigen-presenting molecule) and induces WT1-reactive CD8^+^ (cytotoxic) T-cells^38^. Furthermore, studies in HLA-A*0201-expressing transgenic mice confirmed that the WT2725 injection mixed with the novel W/O pre-emulsion induced HLA-A*0201-restricted, WT2725 peptide-specific CTLs. As observed with other WT1 peptide vaccines^39,40^, injection-site reactions were dose-limiting in preclinical toxicology studies.

Here, we report the results of a phase I study of WT2725. The WT2725 dosing emulsion was administered as a monotherapy to patients with advanced malignancies (glioblastoma, AML, NSCLC, and ovarian cancer) known to overexpress the WT1 protein in the majority of patients^3,4,41^. The study was conducted to determine dose levels for use in future clinical studies, and to evaluate safety, tolerability, and clinical and immunological responses.

## Methods

### Study design

This was a phase I, open-label, dose-escalation and expansion two-part study to define the maximum tolerated dose (MTD), and to evaluate the safety and tolerability of the WT2725 dosing emulsion in adults with advanced malignancies known to overexpress WT1 (registered 18/06/2012, clinicaltrials.gov NCT01621542).

A rolling-six design^42^ was used for enrollment into dose-escalation cohorts until the MTD was reached. When the MTD was reached, up to three expanded cohorts of 10 patients each were allowed, to investigate outcomes in patients with specific tumor types. In part 1, patients were treated with 0.3, 0.9, 3, or 9 mg WT2725 dosing emulsion monotherapy, subcutaneously once every week for 4 weeks (induction phase), then once every 2 weeks for 6 weeks (consolidation phase), and once every 4 weeks thereafter (maintenance phase) until progression or another discontinuation event (Online Resource Fig. 1). Each dose was administered at a single injection site. Since no MTD was established in part 1, the protocol was amended to add part 2, which used a dose- and frequency-intensified treatment schedule. In part 2, patients were treated with 18 or 27 mg WT2725 dosing emulsion monotherapy, subcutaneously once every week for 8 weeks (induction phase), then once every 2 weeks for 10 weeks (consolidation phase), and once every 4 weeks thereafter (maintenance phase) until progression or another discontinuation event (Online Resource Fig. 1). Each dose of the WT2725 dosing emulsion was administered at two injection sites. When possible, sites surrounding the regional lymph nodes in the upper arm, lower abdomen, or femoral area were selected; rotation of injection sites was permitted.

This open-label study involved no randomization or blinding; sequential cohorts were treated according to the dose-escalation scheme and stopping criteria. The study was conducted over ~ 5 years (from July 2012 to May 2017) at six clinical sites in the USA (Houston TX—2 sites, Tucson AZ, La Jolla CA, Chicago IL, Hershey PA). Sample size was based on clinical and practical considerations for this phase I dose-escalation study using the rolling-six design, and was outside of statistical considerations.

### Patients

Patients with advanced-stage, measurable malignancies (that commonly overexpress the WT1 protein: glioblastoma, AML, NSCLC, or ovarian cancer), progressive or recurrent despite standard therapy, or for whom no standard therapy existed, were eligible to participate in part 1 of the study. Determination of WT1 expression was not assessed prior to patient enrollment, however, access to an archival tumor tissue sample or agreement to undergo biopsy after confirmation of study eligibility was required to enable subsequent evaluation of WT1 expression. Other major inclusion criteria were: age ≥ 18 years; Eastern Cooperative Oncology Group Performance Status score of 0–2; HLA-A*0201^+^ and/or HLA-A*0206^+^; adequate bone marrow and immune reserve (absolute neutrophil count ≥ 1000/µL; platelet count ≥ 10 × 10^4^/µL, or ≥ 5 × 10^4^/µL after stem cell transplant; hemoglobin ≥ 9 g/dL; and absolute lymphocyte count ≥ 1000/µL, or ≥ 500/µL after stem cell transplant); adequate renal function (serum creatinine ≤ 1.5 × the upper limit of normal); and adequate hepatic function (total bilirubin ≤ 2.0 mg/dL, or ≤ 3 mg/dL for patients with known Gilbert’s syndrome; and alanine aminotransferase and aspartate aminotransferase ≤ 3 × the upper limit of normal). Patients with glioblastoma or AML (including those who participated in part 1) were eligible to participate in part 2 of the study.

### Endpoints

The primary endpoints were to determine the MTD of the WT2725 dosing emulsion, based on the incidence of dose-limiting toxicities (DLT), and to evaluate the overall safety profile.

DLT were defined as any ≥ grade III adverse events (AEs) that occurred after administration of the first dose of the WT2725 dosing emulsion but before receiving the fifth dose (Days 1–29), not related to underlying disease, intercurrent illness, or concomitant medications. Repeat assessment was required to confirm changes (a shift by ≥ 2 grades) in hematological parameters. Grade III AEs of nausea, vomiting, and fatigue (which are common and manageable in cancer patients) were not considered DLT if they could be reduced to < grade III with standard supportive care.

Secondary endpoints included: proportion of patients in each response category [based on immune-related (ir) response criteria for solid tumors, modified International Working Group response criteria in AML, and/or tumor markers]; and immune response evaluated by induction of WT1-specific CTLs in peripheral blood.

Progression-free survival (PFS) and overall survival (OS) in all patients and by malignancy type were exploratory endpoints.

### Assessments and analyses

Safety assessments, efficacy assessments and analyses are described in Online Resource 1.

### Ethics approval

This study was conducted in accordance with local laws and regulations, the protocol, International Council for Harmonisation Good Clinical Practice, International Council for Harmonisation guidelines, and in alignment with the ethical principles of the Declaration of Helsinki. The study protocol was approved by the UCSD Human Research Protections Program and the Penn State Health Milton S. Hershey Medical Center-Human Subjects Protection Office, and by the Institutional Review Boards at MD Anderson Cancer Center, Western, The University of Chicago, and The University of Texas, MD Anderson Cancer Center, before enrollment of patients into the study at each site.

### Consent to participate

All patients provided informed consent.

### Consent for publication

Not applicable. Personal identifying information are not disclosed and are removed before the use and publication of data.

## Results

### Patients

There were 62 patients in the safety population (part 1 n = 44, part 2 n = 18, Online Resource Fig. 2) and 52, 52, and 61 patients in the DLT, efficacy, and CTL populations, respectively.

In part 1, 44 patients were sequentially allocated to four doses: WT2725 dosing emulsion 0.3 mg (n = 5), 0.9 mg (n = 5), 3 mg (n = 6), and 9 mg (n = 28). In part 2, 18 patients were allocated to two doses: 18 mg (n = 9) and 27 mg (n = 9).

Demographics, baseline characteristics, and malignancy type are shown in Table 1. Median age was 62 years (range 26–76), 61.3% of patients being younger than 65 years. All patients had advanced-stage malignancies, and all but two had an Eastern Cooperative Oncology Group Performance Status of 0 or 1 at baseline. No differences in baseline demographic or clinical characteristics across dose cohorts were expected to influence the results of the study.

**Table 1:** Demographics, patient characteristics, and cancer history at baseline.

	Safety population n = 62
Males, n (%)	29 (46.8)
**Race/ethnicity, n (%)**	
White	53 (85.5)
Black or African American	4 (6.5)
Asian	2 (3.2)
Other	3 (4.8)
Hispanic or Latino	4 (6.5)
Age, years, median (range)	62.0 (26–76)
Weight, kg, median (range)	73.7 (50.3–127.4)
Height, cm, median (range)	167.6 (145.5–188.0)
Body mass index, kg/m^2^, median (range)	26.6 (18.5–46.8)
**ECOG performance status, n (%)**	
0	24 (38.7)
1	36 (58.1)
2	2 (3.2)
Concomitant dexamethasone, n (%)	18 (29.0)
**Malignancy type, n (%)**	
Glioblastoma	20 (32.3)
Ovarian cancer	21 (33.0)
Acute myeloid leukemia	12 (19.4)
Non-small-cell lung cancer	7 (11.3)
Other	2 (3.2)

*ECOG* Eastern Cooperative Oncology Group.

### Safety

No DLT were observed (maximum dose 27 mg WT2725 dosing emulsion). The WT2725 dosing emulsions were well tolerated. Following completion of the planned dose escalation, the sponsor terminated the study for reasons not related to safety. Therefore, the MTD of the WT2725 dosing emulsion was not reached.

Most patients experienced one or more treatment-emergent AEs (TEAEs) during the study (Table 2). Approximately one-third of patients (31.0%) had a ≥ grade III TEAE, and approximately half (53.5%) had a TEAE that the investigator determined as possibly, probably, or definitely related to the study drug; nearly all TEAEs determined as related to study drug were injection-related reactions (Table 2). Serious AEs occurred in approximately one-quarter (23.9%) of patients. Four patients (5.6%) discontinued due to a TEAE (assessed by the investigator as not related to study drug) and no patients died due to causes not related to disease. One death occurred during the study due to disease progression; the death was not reportable as a TEAE, as it was related to progression of the malignancy being treated in the study.

**Table 2:** Incidence of TEAEs.

TEAE category, n (%)	Total, n = 71			
Any TEAE	67 (94.4)			
Any grade ≥ III TEAE	22 (31.0)			
Any treatment-related^a^ TEAE	38 (53.5)			
Any grade ≥ III treatment-related TEAE	1 (1.4)			
Any TEAE with outcome of death	0			
Any SAE	17 (23.9)			
Any treatment-related SAE	1 (1.4)			
Any TEAE leading to treatment discontinuation	4 (5.6)			

	Overall	Grade III	Grade IV/V	Treatment-related
**Individual TEAEs**				
**Injection-site erythema**	**14 (19.7)**	**0**	**0**	**14 (19.7)**
Constipation	13 (18.3)	0	0	0
Nausea	12 (16.9)	0	0	3 (4.2)
Decreased appetite	10 (14.1)	0	0	2 (2.8)
Vomiting	9 (12.7)	0	0	1 (1.4)
Cough	9 (12.7)	0	0	1 (1.4)
**Injection-site pain**	**8 (11.3)**	**0**	**0**	**8 (11.3)**
Hyponatremia	8 (11.3)	3 (4.2)	0	0
Headache	8 (11.3)	0	0	1 (1.4)
Dyspnea	8 (11.3)	1 (1.4)	0	0
**Injection-site reaction**	**7 (9.9)**	**0**	**0**	**7 (9.9)**
Fall	7 (9.9)	0	0	0
Dizziness	7 (9.9)	0	0	0
Anemia	6 (8.5)	3 (4.2)	0	0
Hypokalemia	6 (8.5)	1 (1.4)	0	0
Leukocytosis	5 (7.0)	3 (4.2)	0	0
Diarrhea	5 (7.0)	1 (1.4)	0	0
Pyrexia	5 (7.0)	1 (1.4)	0	0
Asthenia	4 (5.6)	0	0	0
Contusion	4 (5.6)	0	0	1 (1.4)
Hyperglycemia	4 (5.6)	1 (1.4)	0	0
Hypoalbuminemia	4 (5.6)	0	0	0
Dysgeusia	4 (5.6)	0	0	0
Rash	4 (5.6)	1 (1.4)	0	3 (4.2)
Hyperphosphatemia	3 (4.2)	0	0	0
Muscular weakness	3 (4.2)	0	0	0
Pneumonia	2 (2.8)	2 (2.8)	0	0
Vasogenic cerebral edema	2 (2.8)	0	0	0
Confusional state	2 (2.8)	0	0	0
Upper-airway cough syndrome	2 (2.8)	0	0	1 (1.4)

Injection-related reactions are in bold font.

*SAE* serious adverse event, *TEAE* treatment-emergent adverse event.

^a^Assessed as possibly, probably, or definitely related to study drug.

Injection-related reactions were the most frequently reported TEAEs (Table 2, bold font): injection-site erythema 19.7%, injection-site pain 11.3%, injection-site reaction 9.9%. The majority were grade I and none were grade III or dose limiting; grade II injection-related reactions with itching, erythema, pain, and bruising and/or swelling were reported in three patients. No patients discontinued the study due to an injection-related reaction.

### Efficacy

#### Survival

Median PFS was 58 (95% CI 56–81) days (~ 2 months) across all patients with solid tumors, 58 days (95% CI 56–81) in the NSCLC subgroup, 61.5 days (95% CI 56–112) in the ovarian cancer subgroup, and 59 days (95% CI 27–329) in the glioblastoma subgroup, respectively.

**Figure 1:**
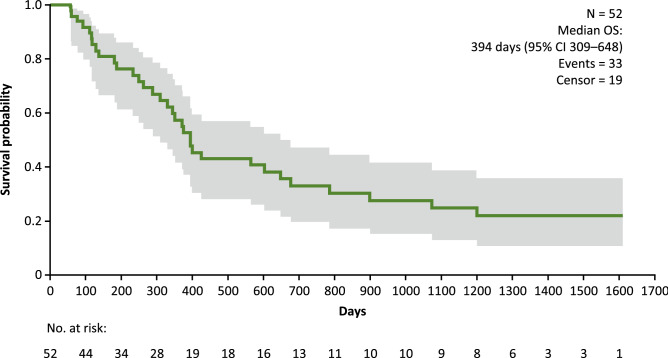
OS (efficacy population). All malignancy types combined. The shaded area represents the 95% CI of the survival probability at that day. *CI* confidence interval, *OS* overall survival.

**Figure 2:**
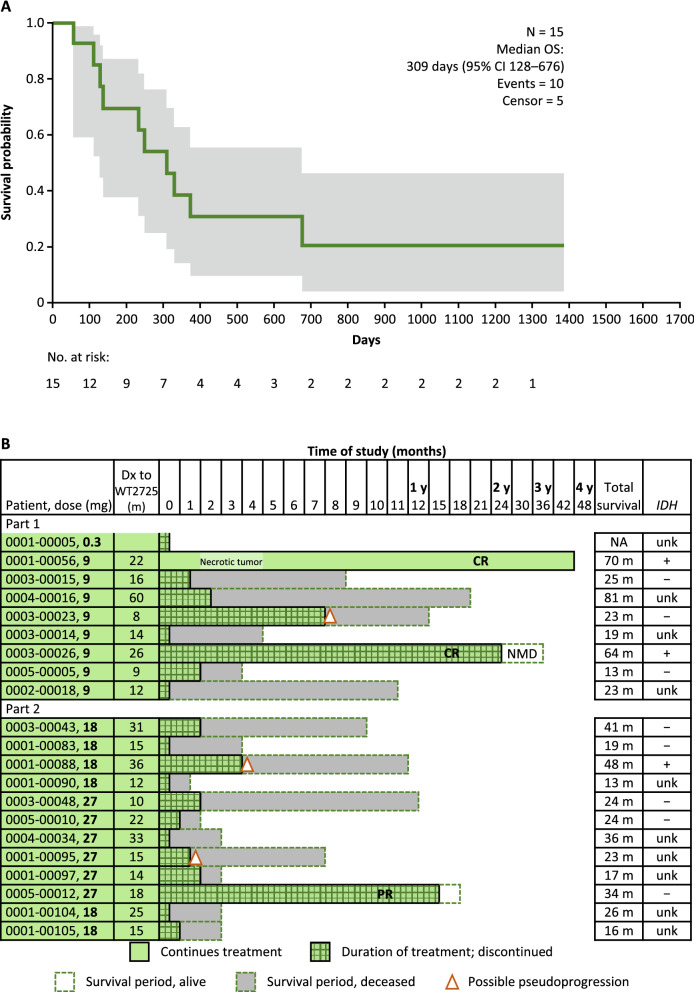
Patient survival in the glioblastoma subgroup. (**A**) OS. The shaded area represents the 95% CI of the survival probability at that day. (**b**) Individual patient survival. *CI* confidence interval, *CR* complete response, *Dx* diagnosis, *IDH* isocitrate dehydrogenase, *m* months, *NMD* no measurable disease, *OS* overall survival, *PR* partial response, *unk* unknown, *y* years.

Overall, 63.5% of study participants died. Median OS was 394 days (13.0 months, 95% CI 309–648 days) across all patients (Fig. 1), 647 days (21.3 months, 95% CI 59 days–not calculable) in the AML subgroup, 275 days (9.0 months, 95% CI 117–351 days) in the NSCLC subgroup, 602 days (19.8 months, 95% CI 344–1200 days) in the ovarian cancer subgroup, and 309 days (10.2 months, 95% CI 128–676 days) in the glioblastoma subgroup (Fig. 2A, glioblastoma only).

One-third (n = 7, 33.3%) of glioblastoma patients survived for ≥ 1 year (Fig. 2B), three (14.3%) for ≥ 18 months, and two (9.5%) for ≥ 2 years. Both patients who survived for ≥ 2 years were in complete radiologic remission, although one discontinued before study termination. At time of analysis, two glioblastoma patients remained on treatment with WT2725 dosing emulsion; one had a complete response (CR) with no measurable disease for > 3 years, and one had a partial response (PR) after > 13 months of treatment.

#### Immune-related tumor response in solid tumor patients

Overall response rate (irCR + irPR) was 7.5% (95% CI 2.6–19.9). Two patients (5.0%) achieved an irCR; both were in the 9 mg dose cohort and the glioblastoma subgroup. One patient (2.5%) achieved an irPR; this patient was in the 27 mg dose cohort and the glioblastoma subgroup.

Ir-stable disease (irSD) was achieved in 12 of 40 evaluable patients (30.0%; 0.3 mg 5.0%, 0.9 mg 2.5%, 3.0 mg 5.0%, 9.0 mg 15.0%, 18 mg 2.5%). Disease control (irCR + irPR + irSD) was achieved in 15 of 40 patients (37.5%).

**Figure 3:**
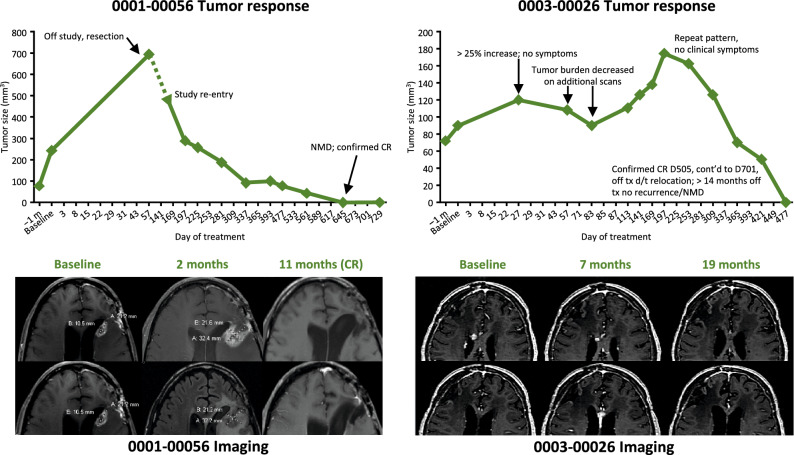
Change in tumor size over time and imaging for the two glioblastoma patients who achieved an immune-related complete response. *CR* complete response, *D* day, *m* month, *NMD* no measurable disease, *tx* treatment.

Response was most prominent in the glioblastoma subgroup. Two of 15 patients achieved irCR, one irPR, and two irSD. The time of maximum response was ~ 16 months (477 days). Figure 3 details tumor size over time (including imaging) for the two glioblastoma patients who achieved an irCR. Patient 0001-00056 underwent surgery for apparent tumor progression at Day 57. The apparent progression was in fact an immune response; the patient re-entered the study and experienced a delayed response. Patient 003-00026 appeared to have tumor progression (on imaging) between days 27 and 197. Treatment was continued and the immune response resolved on its own; a bimodal response was observed in this patient.

There were no irCRs or irPRs in the ovarian cancer subgroup, but nine of 18 patients (50%) had irSD. Overall, 23 patients (57.5%) did not respond to treatment during the course of the study and were reported as either irPD (15.0%) or unconfirmed irPD (42.5%). An additional two patients (5.0%) were considered not evaluable.

#### Modified International Working Group response in AML

Four of nine evaluable patients (44.4%, 95% CI 18.9–73.3) achieved CR (cytogenic responses: 9 mg, n = 2; morphologic responses: 18 mg, n = 2). One additional patient in the 9 mg cohort achieved CR with persistence of cytopenias. Three patients were non-evaluable, due to either a missing baseline or on-treatment data point.

#### Tumor markers

None of the 19 evaluable ovarian cancer patients achieved a biological response (defined as a ≥ 50% reduction from baseline in CA-125 levels). Non-response/non-PD was reported for seven (36.8%) patients.

In AML patients, blood levels of the *WT1* transcript decreased by 0.4% (mean % change from baseline, SD 2.47, range − 5.6 to 4.2) between baseline and maximum on-study measurement (n = 10), and by 1.7% (mean, SD 2.49, range − 5.6 to 0) between baseline and end of study (n = 5). Bone marrow levels of the *WT1* transcript decreased by 1.4% (mean, SD 1.00, range − 2.1 to − 0.7) between baseline and maximum on-study measurement (n = 2).

#### CTL induction

Overall, 62.3% of patients were considered CTL responders. The proportion of CTL responders increased with increasing dose, ranging from 0 in the 0.3 mg cohort to 88.9% in the 27 mg cohort. Representative baseline and post-baseline flow cytometry profiles from one individual are shown in Online Resource Fig. 3.

**Figure 4:**
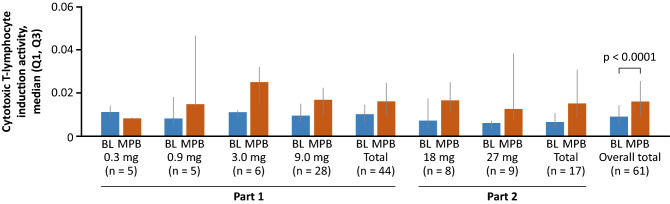
Baseline and post-baseline^a^ CTL induction activity. All malignancy types combined. The CTLs in blood samples were measured by tetramer assay using flow cytometry; the evaluation of CTL induction is defined in the Efficacy assessments section of the Online Resource. P-values were calculated using the Wilcoxon signed-rank test (comparing post-baseline with baseline assessments). ^a^Based on the mean of all post-baseline assessments of each patient. *BL* baseline, *CTL* cytotoxic T-lymphocyte, *MPB* mean of post-baseline.

Mean (± standard deviation [SD]) CTL induction activity at baseline was 0.0228% ± 0.0739 and median CTL induction activity at baseline was 0.0090. For the mean of all post-baseline assessments of each patient, mean (SD) CTL induction activity was 0.0252% ± 0.0387 and median CTL induction activity was 0.0161 (Wilcoxon signed rank test for difference vs 0.0090 at baseline: p < 0.0001) (Fig. 4). When maximum post-baseline assessment values of each patient were assessed, mean (SD) CTL induction activity was 0.0827% ± 0.1776 and median CTL induction activity was 0.0290 (Wilcoxon signed rank test for difference vs 0.0090 at baseline: p < 0.0001).

## Discussion

Subcutaneous injection with WT2725 dosing emulsion (0.3–27 mg) was generally well tolerated and had an acceptable safety profile in adult patients with advanced-stage malignancies known to overexpress the WT1 protein. An MTD could not be established, as no DLT were reported at any of the doses evaluated.

The frequency of the HLA-A*0201 allele ranges between ~ 2% and 44% across the USA; the frequency of the HLA-A*0206 allele is slightly lower (~ 3.2–16.5%)^43^. Therefore, WT2725 could be a feasible treatment option for a substantial proportion of patients (HLA-A*0201^+^ and/or HLA-A*0206^+^) in the USA.

After the initial dose-escalation phase of the study (part 1) and the subsequent dose expansion was completed without DLT, the protocol was amended to include two additional dose-escalation and expansion cohorts of 18 and 27 mg (part 2). These cohorts were restricted to patients with glioblastoma and AML, in order to accumulate more data in these types of malignancy, which typically have relatively high frequencies of *WT1* expression.

Most patients had TEAEs during this study. As in previous clinical studies of WT1 peptide vaccines with the same peptide sequence as WT2725 (administered with various adjuvants and combination therapies)^23,39,40,44–46^, injection-related reactions were the most frequently reported type of TEAE. Most injection-related reactions were grade I, none were grade ≥ III, and no patients discontinued the study due to an injection-related reaction. Overall, approximately one-third of patients had a grade ≥ III TEAE, and approximately half had a TEAE judged as related to study drug; nearly all TEAEs related to study drug were injection-related reactions.

Immune-related tumor responses were most prominent in the glioblastoma subgroup, in which durable objective responses were observed. This finding is consistent with previous case reports and results of small studies, in which WT1 vaccine therapy produced clinical and immunological responses and improved clinical manifestations and quality of life in patients with glioblastoma and glioma^28,47–50^, warranting further investigation in this subgroup of patients. We therefore reported efficacy outcomes for the entire immune-related response criteria population, and separately for the glioblastoma subgroup, to provide more detail with regards to this malignancy type. PFS was comparable across solid tumor subgroups. PFS was 58 days in the full population and 59 days in the glioblastoma subgroup. OS was 394 days in the full population and 647, 275, 602, and 309 days in the AML, NSCLC, ovarian cancer, and glioblastoma subgroups, respectively. Three glioblastoma patients (14%) survived for ≥ 18 months, and two (10%) for ≥ 2 years. Both patients who survived for ≥ 2 years were in complete radiologic remission. As the study was conducted prior to the most recent World Health Organization Classification of Central Nervous System Tumors update, mutational status of isocitrate dehydrogenase was tested retrospectively.

There are challenges associated with monitoring disease progression during treatment with immune therapies^51,52^. Imaging of malignancies in patients treated with immune therapies can detect delayed responses, transient tumor enlargement, and the appearance of new lesions. We therefore used immune-related response criteria to assess response to WT2725 dosing emulsion in this study.

The overall immune-related tumor response rate was 7.5% among 40 evaluable patients. Two patients (5%) achieved an irCR; both were in the glioblastoma subgroup and receiving 9 mg WT2725 dosing emulsion. An irPR was achieved by 2.5% of patients, 30.0% achieved irSD, and 37.5% achieved disease control. Overall, 57.5% of patients did not respond to treatment during the course of the study and were reported as either irPD (15.0%) or unconfirmed irPD (42.5%). In the glioblastoma subgroup, 13.3% achieved irCR, 6.7% irPR, and 13.3% irSD.

Delayed response was noted in glioblastoma patients who responded. In the irCR + irPR population, time of maximum response was at ~ 16 months (477 days). One patient (Patient 0003-00026, glioblastoma treated with 9 mg WT2725 dosing emulsion) had a bimodal response with initial radiographic worsening followed by improvement, followed by recurrent worsening and improvement without any change in management. The driver for this type of radiographic response pattern is unknown. As our understanding of potential biomarkers for response to immunotherapies becomes refined^53^, prospective evaluation of specific biomarkers across various immunotherapeutic modalities will be of value. Furthermore, these two patients exhibited pseudoprogression between days 27 and 197. The RANO brain imaging criteria have recently been modified to take this into account^54^, and are currently being used in a trial of WT1 in glioblastoma (NCT03149003).

WT2725 dosing emulsion stimulated immune activation in 62% of patients, as evidenced by CTL response. CTL response increased with increasing WT2725 dose.

The clinical outcomes, tumor responses and biological marker data observed in our study confirm the promising results observed in other studies of WT1 vaccines^2,23–36,55^. Overall, these data suggest that WT1 vaccines are a potential future treatment option for patients with advanced malignancies, thus providing a rationale for the future clinical development of WT1 vaccines such as WT2725.

Limitations of our study include those typically associated with early phase studies, including the relatively small sample size (further divided into multiple types of advanced malignancy) and the lack of a comparator. However, the purpose of the study was achieved; we concluded that the WT2725 dosing emulsion was well tolerated. In combination with promising initial efficacy data, this study provides a solid foundation for the future development of WT2725.

A general limitation is that vaccination only targets one component of the immune response, and thus may not be effective if patients have deficiencies in other components. Therefore, future directions for the development of effective WT1-targeted treatments for advanced malignancies might involve WT1 vaccine delivery through RNA vaccines or the oncolytic virus system, potentially in combination with other immune therapeutics involving antigen presenting cells, immune checkpoint blockade, natural killer cells, or T cell activation. CAR-T cell therapy targeting WT1 could be another promising direction for the treatment of advanced malignancies.

## Conclusions

WT2725 dosing emulsion was well tolerated in this first-in-human study. Our preliminary tumor response and biological marker data suggest that WT2725 dosing emulsion may exert antitumor activity in malignancies known to overexpress the WT1 protein, particularly glioblastoma, and provide a rationale for future clinical development.

## Supplementary Information

Supplementary Information.

## Data Availability

Sunovion Pharmaceuticals Inc. is part of a clinical trial data-sharing consortium that facilitates access for qualified researchers to selected anonymized clinical trial data. For up-to-date information on data availability please visit: https://www.clinicalstudydatarequest.com/Study-Sponsors.aspx and click on Sunovion.
