# Supplementary material for: A phase I study of the WT2725 dosing emulsion in patients with advanced malignancies
Source: Sci Rep. 2021 Nov 16;11:22355. doi: 10.1038/s41598-021-01707-3 (PMC8595891; doi:10.1038/s41598-021-01707-3)
Supplement: Supplementary file 1 — Supplementary Information. [file 41598_2021_1707_MOESM1_ESM.docx]

**Online resource**

Article title: A phase I study of the WT2725 dosing emulsion in patients with advanced malignancies

Journal: *Scientific Reports*

**Authors:** Siqing Fu, David E. Piccioni, Hongtao Liu, Rimas V. Lukas, Santosh Kesari, Dawit Aregawi, David S. Hong, Kenichiro Yamaguchi, Kate Whicher, Yi Zhang, Yu-Luan Chen, Nagaraju Poola, John Eddy, David Blum

**Corresponding author:** Siqing Fu, The University of Texas MD Anderson Cancer Center (siqingfu@mdanderson.org)

Table of contents

[**Methods** 3](#_Toc82592513)

[**Online Resource Fig. 1** Dosing schedule during study parts 1 and 2 3](#_Toc82592514)

[**Safety assessments** 3](#_Toc82592515)

[**Efficacy assessments** 3](#_Toc82592516)

[**Analyses** 5](#_Toc82592517)

[**Results** 5](#_Toc82592518)

[**Online Resource Fig. 2** Patient flowchart 5](#_Toc82592519)

[**Online Resource Fig. 3** Representative flow cytometry profiles from one individual at **a** Screening (baseline) and **b** Day 281 (post-baseline), including the gating strategy/method to obtain final results 9](#_Toc82592520)

[**References** 10](#_Toc82592521)

# Methods


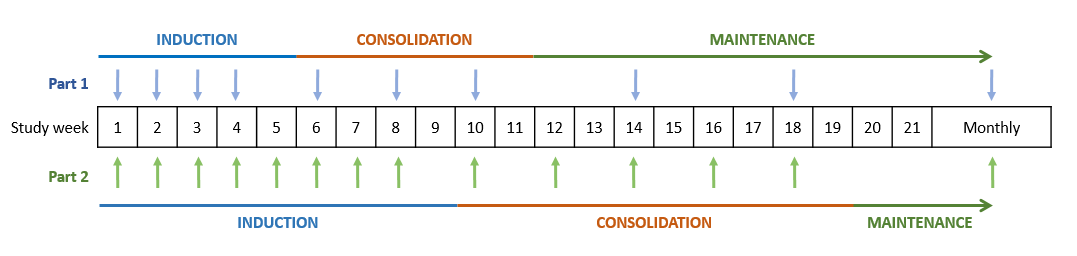


## Online Resource Fig. 1 Dosing schedule during study parts 1 and 2

## Safety assessments

Safety assessments were conducted throughout the study. The incidence of dose-limiting toxicities (DLTs) was evaluated during the DLT evaluation period, which extended from the day of the first dose of WT2725 dosing emulsion to just before the fifth dose (days 1–29); no more than four doses were administered during the DLT evaluation period. An end-of-study assessment was conducted within 28 days of the last dose, before initiating alternative antineoplastic therapy.

All adverse events (AEs) were coded using MedDRA, version 15.0. AEs were graded according to the National Cancer Institute Common Terminology Criteria for Adverse Events, version 4.0. Treatment-emergent AEs were defined as AEs that occurred on or after the first dose of study drug or within 30 days of the last dose. AEs with a missing start date and a stop date on or after the first dose of study drug, or AEs with both a missing start and stop date, were also considered treatment-emergent AEs.

## Efficacy assessments

Progression-free survival was defined as the time from first dose of study drug until date of progression or death due to any cause. Living patients who had not progressed were censored at their date of last contact. Overall survival was defined as the time from first dose of study drug until date of death due to any cause. Living patients were censored at their date of last contact. Survival status was captured after the end of treatment and was considered when determining overall survival.

Solid tumor responses were evaluated according to immune-related response criteria [1, 2]; patients who had at least one tumor assessment 8 weeks after the first dose of study drug, and those who had progressive disease prior to this time point, were evaluable. Responses in Acute Myeloid Leukemia were evaluated according to modified International Working Group response criteria.

Tumor assessments (solid tumors only) were conducted based on total measurable tumor burden via magnetic resonance imaging during screening and every 8 weeks after the first dose of study drug, unless clinical indications required frequent assessment; tumor assessments were first conducted after 8 weeks to allow adequate time for immune activation and consequent antitumor responses. All tumor assessments performed on the day of study drug administration must have been completed before the study drug was given.

Tumor markers CA-125 and WT1 were utilized to assess responses in ovarian cancer and Acute Myeloid Leukemia, respectively. Serum CA-125 and WT1 transcript in blood or bone marrow were measured via quantitative reverse-transcription polymerase chain reaction. Peripheral blood samples were assessed for tumor markers at least every 4 weeks after the first dose of study drug.

Fresh peripheral blood samples were evaluated by tetramer assay using flow cytometry for induction of WT1-specific cytotoxic T-lymphocytes (CTLs). Definitions of CTL responders were: CTLs at least 2× above baseline for at least two post-baseline visits; if fewer than five post-baseline visits: CTLs at least 2× above baseline at one post-baseline visit; if baseline CTLs were 0: post-baseline CTLs must be > 0.

## Analyses

The last non-missing value prior to the first dose of study drug was used as the baseline measurement. Doses were defined as the intended and starting dose to which each patient was enrolled.

Data analysis and summarization were performed for safety, DLT, efficacy, and CTL populations (database locked in October 2017). The safety population included all enrolled patients who received at least one dose of study drug, and was used for the analysis of all safety data, except as noted below. The DLT population comprised all patients in the safety population who experienced a DLT within the DLT evaluation period or who completed the DLT evaluation period without experiencing a DLT. The efficacy population comprised all patients in the safety population who were evaluable for response. The CTL population comprised all patients in the safety population who had a baseline assessment of percentage of WT1^+^ cells among CD8^+^ cells, and at least one post-baseline assessment. P-values were calculated using the Wilcoxon signed-rank test. Statistical analyses were performed using SAS® version 9.4 (SAS Institute, Cary, NC, USA).

# Results


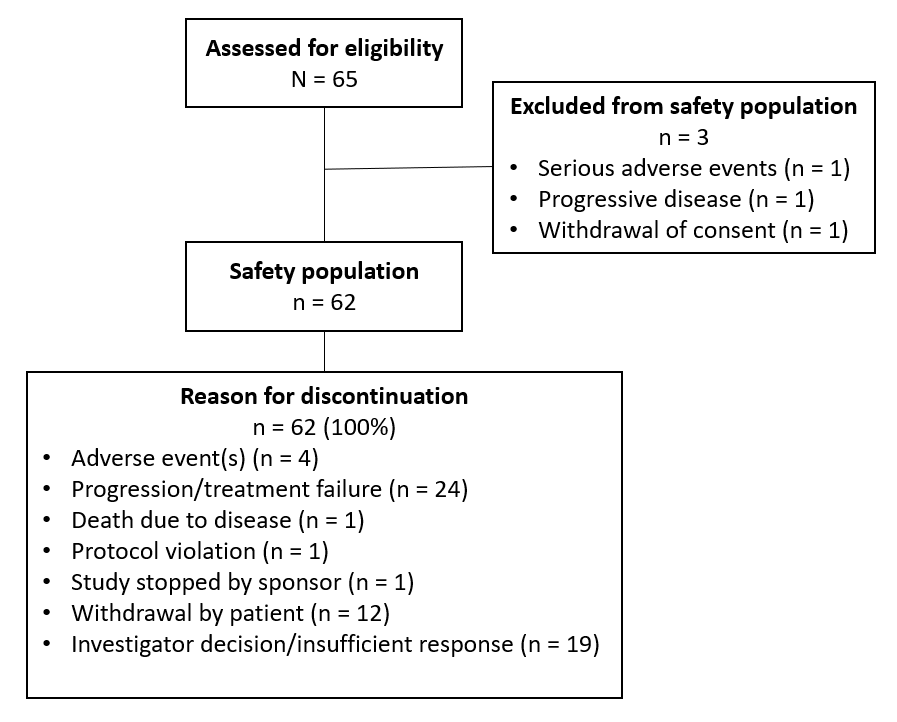


## Online Resource Fig. 2 Patient flowchart

**A** Screening (baseline)
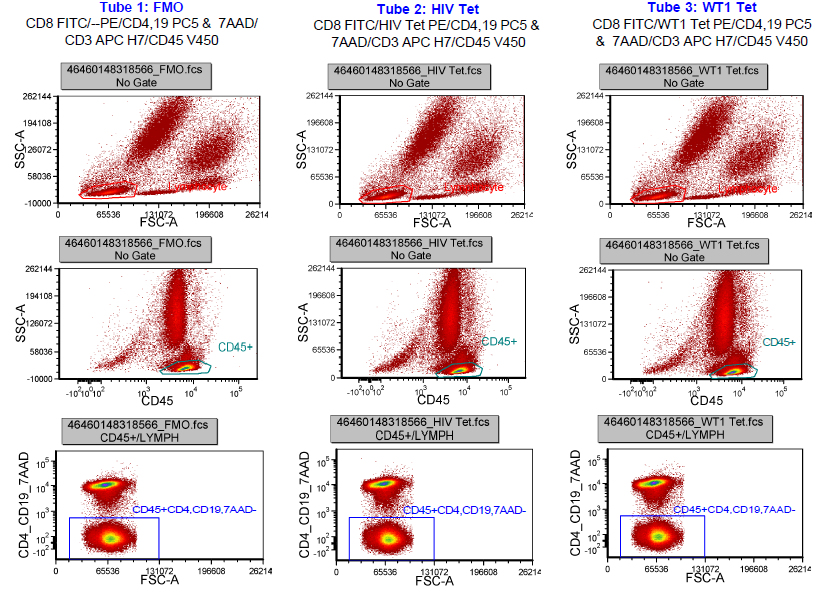

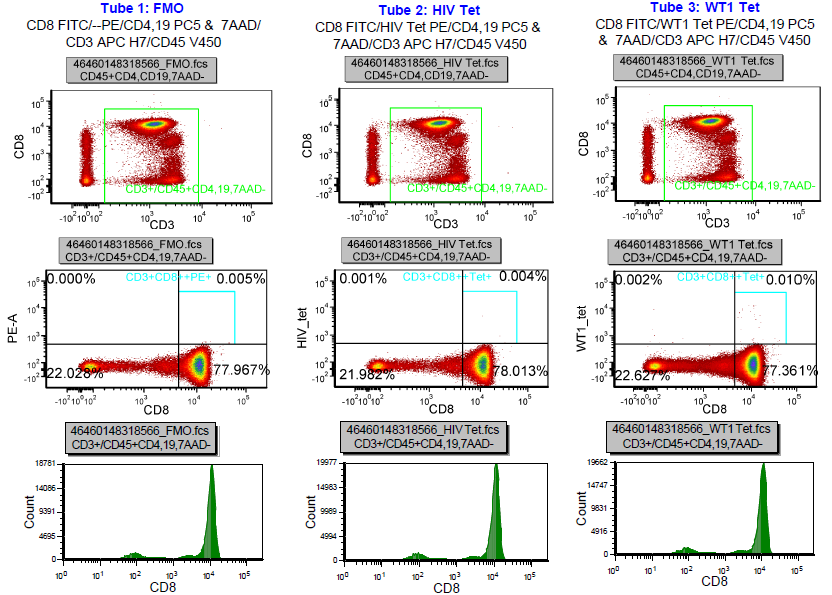

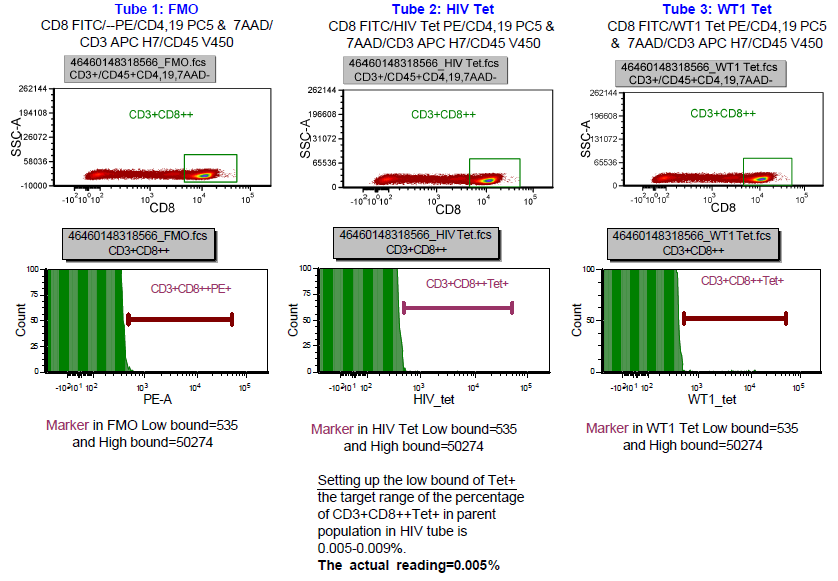

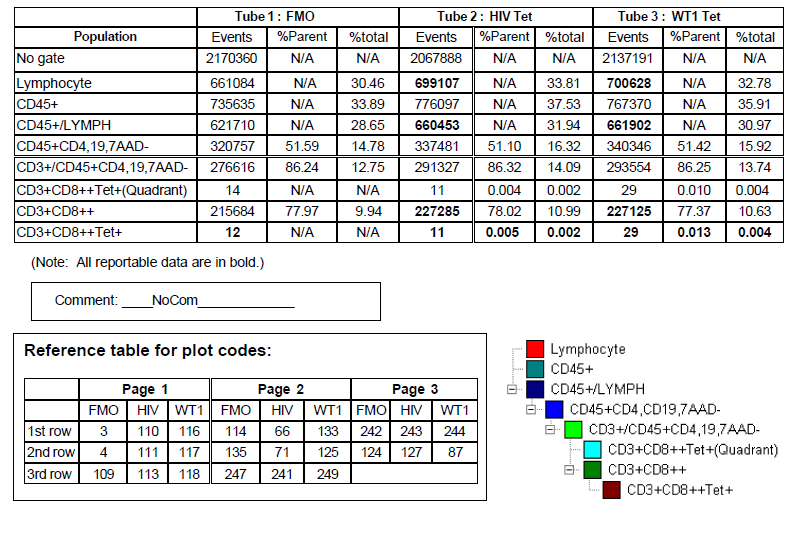


**B** Day 281 (post-baseline)


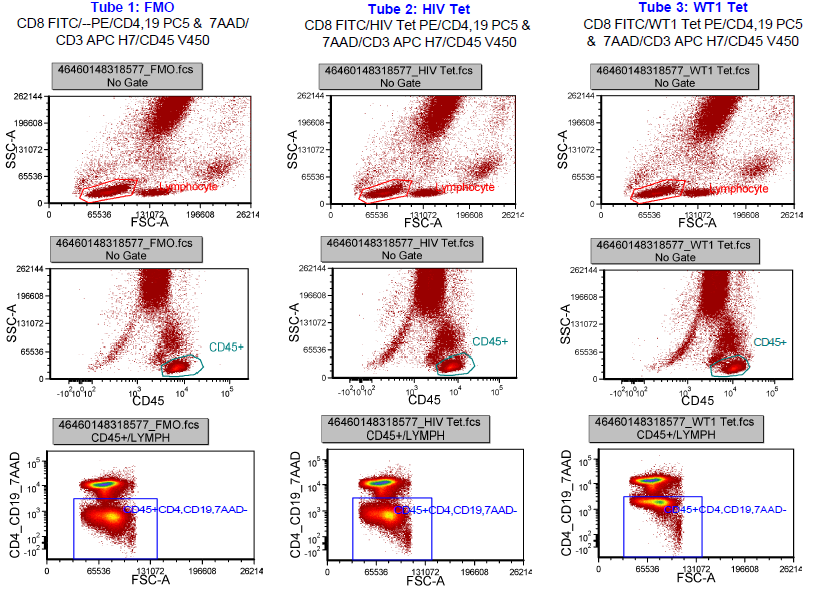

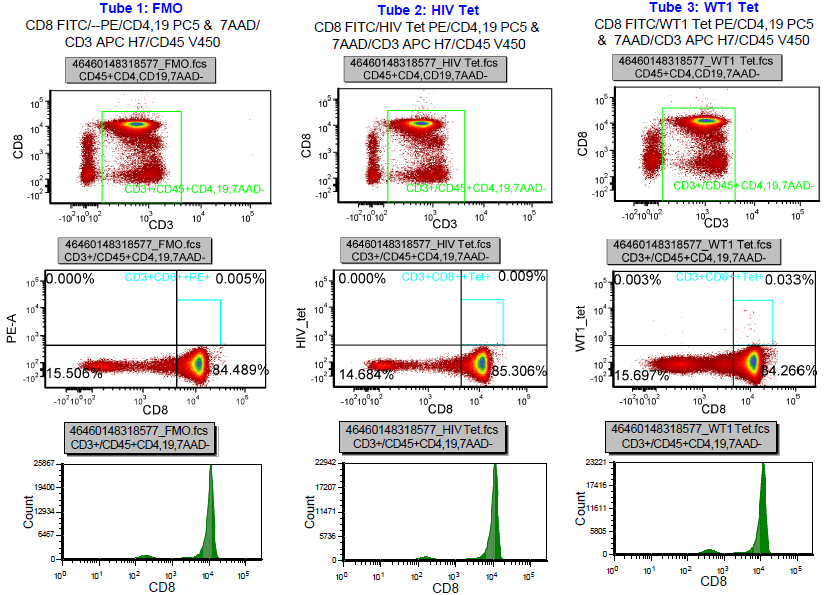

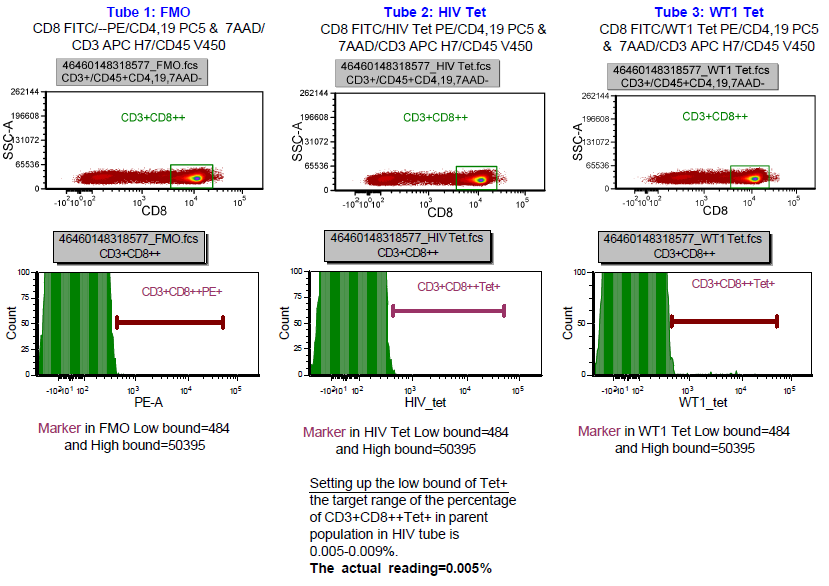

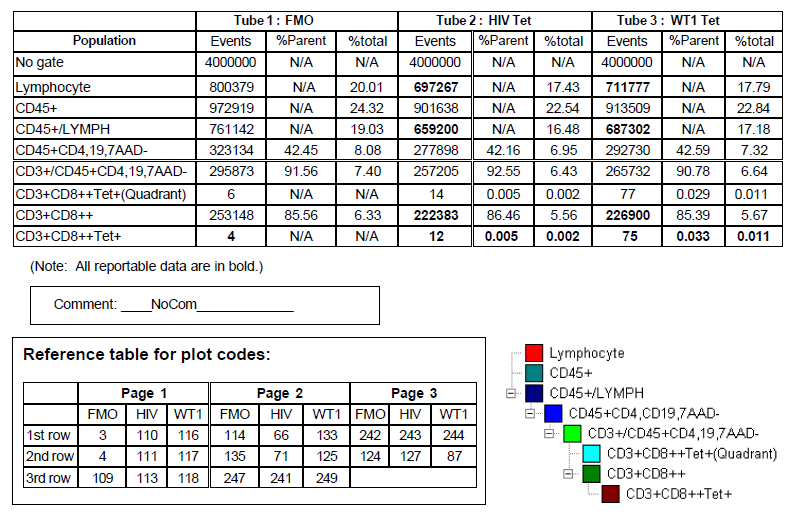


## Online Resource Fig. 3 Representative flow cytometry profiles from one individual at a Screening (baseline) and b Day 281 (post-baseline), including the gating strategy/method to obtain final results

# References

1 Wolchok, J. D. *et al.* Guidelines for the evaluation of immune therapy activity in solid tumors: immune-related response criteria. *Clin. Cancer Res.* **15**, 7412–7420 (2009).

2 Hoos, A. *et al.* Improved endpoints for cancer immunotherapy trials. *J. Natl. Cancer Inst.* **102**, 1388–1397 (2010).
